# On Constructing Informationally Complete Covariant Positive Operator-Valued Measures

**DOI:** 10.3390/e25050783

**Published:** 2023-05-11

**Authors:** Grigori Amosov

**Affiliations:** 1 Steklov Mathematical Institute of Russian Academy of Sciences, ul. Gubkina 8, Moscow 119991, Russia; gramos@mi-ras.ru; 2 Center of Pure Mathematics, Moscow Institute of Physics and Technology, Dolgoprudny 141701, Russia; 3 Saint Petersburg State University, Saint Petersburg 199034, Russia; 4 Institute of Mathematics with Computer Center of the Ufa Science Center of Russian Academy of Sciences, Ufa 450008, Russia

**Keywords:** projective unitary representation of locally compact Abelian group, positive operator-valued measures, informational completeness, the Naimark dilation, coherent states

## Abstract

We study a projective unitary representation of the product $G=\tilde{G}\times G$, where *G* is a locally compact Abelian group and $\hat{G}$ is its dual consisting of characters on *G*. It is proven that the representation is irreducible, which allows us to define a covariant positive operator-valued measure (covariant POVM) generated by orbits of projective unitary representations of G. The quantum tomography associated with the representation is discussed. It is shown that the integration over such a covariant POVM defines a family of contractions which are multiples of unitary operators from the representation. Using this fact, it is proven that the measure is informationally complete. The obtained results are illustrated by optical tomography on groups and by a measure with a density that has a value in the set of coherent states.

## 1. Introduction

The theory of noncommutative operator-valued measures, dating back to Naimark’s pioneering work [1], has found many applications in quantum information theory. Each such object, named a positive operator-valued measure (POVM), is defined on some measurable space *X*, takes values in the cone of positive operators in a Hilbert space, *H*, and is normed by the condition that the measure of the entire space is equal to the identity operator. Thus, it defines the measurement quantum channel, that is, a map from the convex set of quantum states to the set of probability distributions on *X* obtained by taking a trace of the product of a state and a positive operator from the measure [2]. Measurement quantum channels have a special structure and require special research. This is due to the fact that, unlike other quantum channels, the mapping occurs between a quantum system and a classical one, defined by the measurable space *X* on which the measure was set. Meanwhile, if *X* is not discrete (as is the case for continuous variables), a classical system cannot be embedded into a quantum one. Recently, there have been significant works devoted to the calculation of various information characteristics of such hybrid (classical–quantum) systems with POVMs given on a space with continuous variables [3,4].

An important class of POVMs is informationally complete POVMs, that is, those that allow you to restore the state based on the measurement results. For the case of discrete spaces *X* and finite dimensional Hilbert spaces *H*, the most famous examples of informationally complete measures are SIC-POVMs [5] as well as POVMs defined by mutually unbiased bases (MUBs) [6]. The existence of such informationally complete POVMs has been proven for far from all dimensions of *H*. Some kind of duality between SIC-POVMs and MUBs was noted in [7].

For applications in practice, the situation when the space on which the measure is defined is a locally compact Abelian group X=G often occurs. Examples here include homodyne detection (G=R), the phase measurement (G=T) and the spin measurement ($G=Z_{n}$). Since a scalar Haar measure ν is defined on locally compact groups *G*, it is natural to assume that a POVM on *G* has some operator density with repect to the measure ν. Moreover, if a unitary representation of *G* is defined in a Hilbert space *H* in which positive operators of POVM act, we can limit ourselves to searching for measures covariant with respect to this representation. A natural way to construct covariant POVMs is to integrate over orbits of an irreducible projective unitary representation of some group [2,8]. In the case of compact groups, the Haar measure of *G* is finite, resulting in the existence of integral over orbits. If *G* is locally compact only, problems arise with the existence of an integral, which can be solved by using the Pontryagin duality principle [9]. With this approach, it is necessary to consider the direct product of the group itself and the group dual to it according to Pontryagin [10].

The problem of the existence of informationally complete measurements is closely related to quantum tomography, which assumes not one, but a series of measurements determined by a set of different POVMs. In quantum optics, the informationally complete measurement is fulfilled under the heterodyne detection, resulting in the Husimi function of a quantum state [11]. Such a measurement corresponds to the informationally complete measure possessing a density consisting of projectors on coherent states. On the other hand, an optical quantum tomogram (a set of quadrature measurements representing linear combinations of coordinates and momentum) appears as a result of the homodyne detection [12]. The connection between the Husimi function and the optical quantum tomogram is known [13]. Both of these characteristics provide complete information about the quantum state.

In this paper, we would like to develop the ideas of the relationship between the homodyne and heterodyne detections based on formal mathematical theory. In our approach, the role of heterodyne detection will be played by the measurement channel defined by a covariant POVM possessing a projection-valued density, which is the orbit of the action of a projective unitary representation of a locally compact group on a fixed one-dimensional projection. The role of homodyne detection is played by quantum tomography on groups which was already developed in [14] on the basis of the Pontryagin duality principle.

Earlier, we proved that the tomography we introduced to the group allows us to restore the quantum state. Now, we will show that this fact can be used to prove the completeness of the covariant measure we have constructed. The key technique that we use is the study of the set of contraction operators defined by our covariant POVM. Unexpectedly, it turned out that such contractions are multiplies of unitary operators of the representation.

The paper is organized as follows. At first (Section 2), we provide the necessary information about locally compact Abelian groups *G* and the Pontryagin duality. Section 3 is devoted to the construction of projective unitary representation of the direct product of the group *G* and its dual $\hat{G}$ in a Hilbert space. We prove that this representation introduced in [10,14] is irreducible and provide the necessary information about tomography of groups from [10,14]. In Section 4, the construction of a POVM by means of orbits of the projective unitary representation introduced in [14] is extended to the entire space. Then, we define a family of unitary operators associated with our POVM by means of the Naimark dilation. Finally, by reducing the unitary operators, we obtain contractions corresponding to the constructed POVM and show that these contractions are multiples of the unitary operators of the representation. Then, we prove that this results in the informational completeness of the POVM. Section 5 is devoted to an illustrative example, in which a measure generated by coherent states is considered. This is the famous Glauber–Sudarshan measure, which possesses the operator-valued density consisting of projections on coherent states. Section 6 contains the concluding remarks.

## 2. Preliminaries

Throughout this article we will use the results from an abstract harmonic analysis [15]. Let *G* be a locally compact Abelian group with a Haar measure ν which is invariant with respect to shifts ν(B+g)=ν(B) for any measurable set B⊂G and g∈G. A continuous map χ:G→T={z∈C:|z|=1} is said to be a character of *G* if it is a homomorphism χ(gh)=χ(g)χ(h),g,h∈G. The set of all characters $\hat{G}$ equipped with an operation of multiplication $\left[\chi^{\prime }\chi \right]\left(g\right)=\chi^{\prime }\left(g\right)\chi \left(g\right),\;\chi ,\chi^{\prime }\in \hat{G},\;g\in G,$ is said to be a dual group with respect to *G*. It is known that $\hat{G}$ is also a locally compact Abelian group with a Haar measure $\hat{\nu }$ determined uniquely up to a multiplication by a positive constant.

Given $\psi \in L^{1}\left(G,\nu \right)$, we can define the function $\hat{f}$ on $\hat{G}$ determined by the formula

(1) $$\hat{\psi }\left(\chi \right)=\int_{G}\bar{\chi }\left(g\right)\psi \left(g\right)d\nu \left(g\right).$$

The Pontryagin duality [9,15] states that there exists the unique Haar measure $\hat{\nu }$ on $\hat{G}$ such that (1) can be extended to the isometrical map $F:L^{2}\left(G,\nu \right)\to L^{2}\left(\hat{G},\hat{\nu }\right)$, named the (abstract) Fourier transform. Moreover, the inverse map $F^{-1}:L^{2}\left(\hat{G},\hat{\nu }\right)\to L^{2}\left(G,\nu \right)$ has the form

$$F^{-1}\left(\hat{\psi }\right)\left(g\right)=\int_{\hat{G}}\chi \left(g\right)\hat{\psi }\left(\chi \right)d\hat{\nu }\left(\chi \right).$$

Thus, we obtain

(2) $$\int_{\hat{G}\times G}\chi \left(h\right)\bar{\chi (g)}\psi \left(g\right)d\hat{\nu }\left(\chi \right)d\nu \left(g\right)=\psi \left(h\right)$$

with sufficiently smooth ψ. Then, (2) can be extended up to $F^{-1}\circ F\psi =\psi$ for any $\psi \in L^{2}\left(G,\nu \right)$.

## 3. Irreducible Projective Unitary Representation of $\hat{G}\times G$ and Quantum Tomography of Groups

Let $H=L^{2}\left(G,\nu \right)$ and define a set of unitary operators in H by the formula

(3) $$\left[U_{\chi ,g}\psi \right]\left(h\right)=\chi \left(h\right)\psi \left(h+g\right),\;\chi \in \hat{G},\;g,h\in G,\;\psi \in H.$$

The operators (3) are known to form a projective unitary representation of the group $G=\hat{G}\times G$ [10]

(4) $$U_{\chi ,g}U_{\chi^{\prime },g^{\prime }}=\chi^{\prime }\left(g\right)U_{\chi \chi^{\prime },g+g^{\prime }},\;\chi ,\chi^{\prime }\in \hat{G},\;g,g^{\prime }\in G.$$

It immediately follows from (4) that

(5) $$U_{\chi ,g}U_{\chi^{\prime },g^{\prime }}=\chi^{\prime }\left(g\right)\bar{\chi (g^{\prime })}U_{\chi^{\prime },g^{\prime }}U_{\chi ,g}$$

and

(6) $$U_{\chi ,g}^{*}=\chi \left(g\right)U_{\bar{\chi },-g}.$$

**Proposition 1.**  *The Formula (3) determines an irreducible projective unitary representation of $\hat{G}\times G$ in H.*

**Proof.**  Let S(H) be the set of all quantum states (positive unit trace operators) in H equipped with a semiliniear form

(7) $$<<\rho ,\sigma >>=Tr\left(\rho^{*}\sigma \right),\;\rho ,\sigma \in S\left(H\right).$$

Denote $S_{2}\left(H\right)$ as the space of all operators obtained by a completion with respect to the norm corresponding to (7), then $S_{2}\left(H\right)$ is said to be the Hilbert–Schmidt class of operators in H [16]. It is known that the map $T:\;S_{2}\left(H\right)\to L^{2}\left(\hat{G}\times G\right)$ determined by the formula

$$\left[T\rho \right]\left(\chi ,g\right)=Tr\left(\rho U_{\chi ,g}\right),\;\chi \in \hat{G},\;g\in G,$$

establishes an isometrical isomorphism [10]. Moreover, the inverse transformation is given by

$$T^{-1}F=\int_{\hat{G}\times G}F\left(\chi ,g\right)U_{\chi ,g}^{*}d\hat{\nu }\left(\chi \right)d\nu \left(g\right),\;F\in L^{2}\left(\hat{G}\times G\right).$$

Hence, any finite dimensional projection *P* belonging to $S_{2}\left(H\right)$ can be represented as

$$P=\int_{\hat{G}\times G}F_{P}\left(\chi ,g\right)U_{\chi ,g}^{*}d\hat{\nu }\left(\chi \right)d\nu \left(g\right)$$

for some $F_{P}\in L^{2}\left(\hat{G}\times G\right)$. Thus, the claim $U_{\chi ,g}X=XU_{\chi ,g}$ for all $\chi \in \hat{G},\;g\in G$ and a fixed bounded operator *X* in H results in XP=PX for all finite dimensional projections *P*. The result follows. □

Using the ideas in [10], let us define the maximal Abelian subgroups $U_{\chi ,g}\subset U$ including the element ${\left[\chi \left(g\right)\right]}^{1/2}U_{\chi ,g}\in U$. It follows from (5) that

$$\chi^{\prime }\left(g^{\prime \prime }\right)=\chi^{\prime \prime }\left(g^{\prime }\right)\;\mathrm{for}\;\mathrm{any}\;\mathrm{two}\;U_{\chi^{\prime },g^{\prime }},U_{\chi^{\prime \prime },g^{\prime \prime }}\in U_{\chi ,g}.$$

Moreover, for inverse elements (6) we obtain

(8) $${\left({\left[\chi^{\prime }\left(g^{\prime }\right)\right]}^{1/2}U_{\chi^{\prime },g^{\prime }}\right)}^{*}={\bar{|\chi^{\prime }\left(g^{\prime }\right)|}}^{1/2}U_{{\bar{\chi }}^{\prime },g^{\prime }},\;U_{\chi^{\prime },-g^{\prime }}\in U_{\chi ,g}.$$

**Remark 1.**  *Wherever we encounter the need to extract the root from a complex number, we will act according to the rule $e^{i\phi }\to e^{\frac{i\phi }{2}}$.*

Since (8) holds true, $U_{\chi ,g}$ is closed with respect to the operation of taking the inverse and is indeed a group.

**Proposition 2.**  *The function*

$$f_{\rho ,\chi ,g}\left(\chi^{\prime },g^{\prime }\right)={\left[\chi^{\prime }\left(g^{\prime }\right)\right]}^{1/2}Tr\left(\rho U_{\chi^{\prime },g^{\prime }}\right),\;$$

*on the group of elements $\left[\chi^{\prime }\left(g^{\prime }\right)\right]U_{\chi^{\prime },g^{\prime }}\in U_{\chi ,g}$ is positive definite and*

$$f_{\rho ,\chi ,g}\left(\chi^{\prime },g^{\prime }\right)=\int_{\hat{G}\times G}\chi^{\prime \prime }\left(g^{\prime }\right)\chi^{\prime }\left(g^{\prime \prime }\right)d\mu_{\rho ,\chi ,g}\left(\chi^{\prime \prime },g^{\prime \prime }\right)$$

*for some probability measure $\mu_{\rho ,\chi ,g}$ on $\hat{G}\times G$.*

**Proof.**  Indeed, since $U_{\chi ,g}=\left\{{\left[\chi^{\prime }\left(g^{\prime }\right)\right]}^{1/2}U_{\chi^{\prime },g^{\prime }}\right\}\in {\left[\chi \left(g\right)\right]}^{1/2}U_{\chi ,g}$ is an Abelian group, then $f_{\rho ,\chi ,g}$ is a positive definite function on the group $U_{\chi ,g}$, such that

$$f_{\rho ,\chi ,g}\left(0,0\right)=1,$$

$$\sum_{j,k}\lambda_{j}{\bar{\lambda }}_{k}f_{\rho ,\chi ,g}\left(\chi_{j}{\bar{\chi }}_{k},g_{j}-g_{k}\right)=\sum_{j}Tr(\rho |\sum_{j}\lambda_{j}|\chi_{j}\left(g_{j}\right){|}^{1/2}U_{\chi_{j},g_{k}}{|}^{2})\geq 0.$$

Hence, the results follows from the Bochner theorem [15]. □

We shall say a set of all probability distributions $\left\{\mu_{\rho ,\chi ,g},\;\left(\chi ,g\right)\in \hat{G}\times G\right\}$ a quantum tomogram of a state ρ∈S(H). This set of distributions contains the same information about the quantum state as the density operator ρ. In fact, this set allows us to restore the characteristic function [10]

(9) $$f_{\rho }\left(\chi ,g\right)={\left[\chi \left(g\right)\right]}^{1/2}Tr\left(\rho U_{\chi ,g}\right),\;\left(\chi ,g\right)\in \hat{G}\times G$$

that, in turn, gives rise to the density operator ρ [10,14]. The whole point of our activity is to compare such an approach, which allows us to restore the density operator from a set of probability measures determined in Proposition 2 with the measurement of the quantum state using an informationally complete measure.

## 4. The Covariant POVM Generated by the Representation and Its Completeness

Fix a unit vector $\psi_{0}\in H$. Since the representation (3) is irreducible due to Proposition 1, the closure of $span(U_{\chi ,g}\psi_{0},\;\chi \in \hat{G},\;g\in G)$ coincides with H. Denote Σ and $B{\left(H\right)}_{+}$ as the σ-algebra of measurable subsets of $G=\hat{G}\times G$ and the positive cone of all positive bounded operators in H. Then, the map $M:\Sigma \to B{\left(H\right)}_{+}$ determined by the formula

(10) $$M\left(B\right)=\int_{B}|U_{\chi ,g}\psi_{0}\rangle \langle U_{\chi ,g}\psi_{0}|d\hat{\nu }\left(\chi \right)d\nu \left(g\right)$$

is a covariant positive operator-valued measure [10].

Following the Naimark theorem [1], there exists an isometrical embedding H⊂K and a projection-valued measure *E* on G such that

$$M\left(B\right)=P_{H}{E\left(B\right)|}_{H},\;B\in \Sigma ,$$

where $P_{H}$ is the orthogonal projection on H. Any irreducible unitary representation of the group G in a Hilbert space K is one-dimensional and has the form

$$\pi \left(g\right)\psi_{\chi }=\chi \left(g\right)\psi_{\chi },\;\chi \in \hat{G},\;g\in G,\;\phi_{\chi }\in K.$$

This implies that any arbitrary unitary representation of G has the Naimark form [17]

$$\pi \left(\chi ,g\right)=\int_{G}\chi^{\prime }\left(g\right)\bar{\chi (g^{\prime })}dE\left(\chi^{\prime },g^{\prime }\right),\;\left(\chi ,g\right)\in \hat{G}≅G.$$

Hence, any unitary operator in K of the form

$$W_{\chi ,g}=\int_{G}\chi^{\prime }\left(g\right)\bar{\chi (g^{\prime })}dE\left(\chi^{\prime },g^{\prime }\right)$$

determines the contraction $T_{\chi ,g}$ (i.e., an operator of a norm of at most one) in H by means of the formula

$$T_{\chi ,g}=P_{H}W_{\chi ,g}{|}_{H}=$$

(11) $$\int_{G}\chi^{\prime }\left(g\right)\bar{\chi (g^{\prime })}dM\left(\chi^{\prime },g^{\prime }\right)=\int_{G}\chi^{\prime }\left(g\right)\bar{\chi (g^{\prime })}|U_{\chi^{\prime },g^{\prime }}\psi_{0}\rangle \langle U_{\chi^{\prime },g^{\prime }}\psi_{0}|d\hat{\nu }\left(\chi^{\prime }\right)d\nu \left(g^{\prime }\right)$$

It is useful to remark that $W_{\chi ,g}$ is known as a unitary dilation of $T_{\chi ,g}$ and satisfies the relation [18]

$$T_{\chi ,g}^{n}=P_{H}W_{\chi ,g}^{n}{|}_{H},\;n=0,1,2,3,\cdots$$

**Remark 2.**  *Note that a set of contractions $\left(T_{\chi ,g}\right)$ determines a representation for the semigroup $\left\{\left(\chi^{n},ng\right),\;n=0,1,2,3,\cdots \right\}$ due to the property*

$$W_{\chi ,g}^{n}=W_{\chi^{n},ng},\;n=0,1,2,3,\cdots$$

*under a construction.*

**Theorem 1.**  *There exists a complex-valued measurable function f on the group G satisfying the relation $0<|f\left(\chi ,g\right)|\leq 1,\;\chi \in \hat{G},\;g\in G$, such that*

$$T_{\chi ,g}=f\left(\chi ,g\right)U_{\chi ,g},\;\chi \in \hat{G},\;g\in G.$$

**Proof.**  Taking into account (4), (6) and (11), we obtain

$$T_{\chi ,g}U_{\chi^{\prime \prime },g^{\prime \prime }}=\int_{G}\chi^{\prime }\left(g\right)\bar{\chi (g^{\prime })}|U_{\chi^{\prime },g^{\prime }}\psi_{0}\rangle \langle U_{\chi^{\prime \prime },g^{\prime \prime }}^{*}U_{\chi^{\prime },g^{\prime }}\psi_{0}|d\hat{\nu }\left(\chi^{\prime }\right)d\nu \left(g^{\prime }\right)=$$

$$\int_{G}\chi^{\prime }\left(g\right)\bar{\chi (g^{\prime })}\bar{\chi^{\prime \prime }\left(g^{\prime \prime }\right)}\chi^{\prime }\left(g^{\prime \prime }\right)|U_{\chi^{\prime },g^{\prime }}\psi_{0}\rangle \langle U_{\bar{\chi^{\prime \prime }}\chi^{\prime },g^{\prime }-g^{\prime \prime }}\psi_{0}|d\hat{\nu }\left(\chi^{\prime }\right)d\nu \left(g^{\prime }\right)=$$

$$\int_{G}\chi^{\prime }\chi^{\prime \prime }\left(g\right)\bar{\chi (g^{\prime }+g^{\prime \prime })}\bar{\chi^{\prime \prime }\left(g^{\prime \prime }\right)}\chi^{\prime }\chi^{\prime \prime }\left(g^{\prime \prime }\right)|U_{\chi^{\prime }\chi^{\prime \prime },g^{\prime }+g^{\prime \prime }}\psi_{0}\rangle \langle U_{\chi^{\prime },g^{\prime }}\psi_{0}|d\hat{\nu }\left(\chi^{\prime }\right)d\nu \left(g^{\prime }\right)=$$

$$\chi^{\prime \prime }\left(g\right)\bar{\chi (g^{\prime \prime })}U_{\chi^{\prime \prime },g^{\prime \prime }}\int_{G}\chi^{\prime }\left(g\right)\bar{\chi (g^{\prime })}|U_{\chi^{\prime },g^{\prime }}\psi_{0}\rangle \langle U_{\chi^{\prime },g^{\prime }}\psi_{0}|d\hat{\nu }\left(\chi^{\prime }\right)d\nu \left(g^{\prime }\right)=$$

$$\chi^{\prime \prime }\left(g\right)\bar{\chi (g^{\prime \prime })}U_{\chi^{\prime \prime },g^{\prime \prime }}T_{\chi ,g},\;\chi ,\chi^{\prime \prime }\in \hat{G},\;g,g^{\prime \prime }\in G.$$

Since the projective representation (3) is irreducible in H, (5) results in $T_{\chi ,g}=f\left(\chi ,g\right)U_{\chi ,g}$ by the Schur lemma. □

The following statement shows that the measurement fulfilled by (10) is informationally complete.

**Theorem 2.**  *The density of probability distribution*

$$p_{\rho }\left(\chi ,g\right)=\langle U_{\chi ,g}\psi_{0},\rho U_{\chi ,g}\psi_{0}\rangle$$

*allows us to restore a state ρ by the formula*

$$\rho =\int_{G}f^{-1}\left(\chi ,g\right)U_{\chi ,g}^{*}\int_{G}\chi \left(g^{\prime }\right)\bar{\chi^{\prime }}\left(g\right)p_{\rho }\left(\chi^{\prime },g^{\prime }\right)d\hat{\nu }\left(\chi^{\prime }\right)d\nu \left(g^{\prime }\right)d\hat{\nu }\left(\chi \right)d\nu \left(g\right).$$

**Proof.**  At first, notice that

$$\rho =\int_{G}Tr\left(\rho U_{\chi ,g}\right)U_{\chi ,g}^{*}d\hat{\nu }\left(\chi \right)d\nu \left(g\right)$$

In fact, the map $\rho \to Tr(\rho U_{\chi ,g})$ determines the isometrical isomorphism [10,14]

$$Tr\left(\rho \bar{\sigma }\right)=\int_{G}Tr\left(\rho U_{\chi ,g}\right)\bar{Tr(\sigma U_{\chi ,g})}d\hat{\nu }\left(\chi \right)d\nu \left(g\right).$$

This implies that

$$\int_{G}Tr\left(\rho U_{\chi ,g}\right)Tr\left(U_{\chi ,g}^{*}|\xi \rangle \langle \eta |\right)d\hat{\nu }\left(\chi \right)d\nu \left(g\right)=$$

Tr(ρ|η〉〈ξ|)=〈ξ,ρη〉.

Now, it follows from Theorem 1 that

$$Tr\left(\rho U_{\chi ,g}\right)=f^{-1}\left(\chi ,g\right)\int_{G}\chi^{\prime }\left(g\right)\bar{\chi (g^{\prime })}\langle U_{\chi^{\prime },g^{\prime }}\psi_{0},\rho U_{\chi^{\prime },g^{\prime }}\psi_{0}\rangle d\hat{\nu }\left(\chi^{\prime }\right)d\nu \left(g^{\prime }\right).$$

□

## 5. The Optical Quantum Tomography and the Measure with Density Taking Values in Coherent States

In homodyne detection, the quadrature components of the electromagnetic field are measured [12]. Observables corresponding to this measurement are $\cos\varphi \hat{x}+\sin\varphi \hat{p},\;0\leq \varphi <2\pi$, where $\hat{x}$ and $\hat{p}$ play the role of coordinates and momentum operators. Quadrature components can be determined if the field mode under study interferes with another reference beam of the same frequency and well-defined phase φ. In this section, we will explain how the abstract mathematical apparatus introduced previously works in quantum optics.

Consider G=R giving $\hat{G}=R$ and $G=\hat{G}\times G=R^{2}$. Now, $H=L^{2}\left(R\right)$ and

$$\left(U_{x,y}\psi \right)\left(t\right)=e^{itx}\psi \left(t+y\right),\;\left(x,y\right)\in R^{2},\;\psi \in H.$$

Then,

$$U_{x_{1},y_{1}}U_{x_{2},y_{2}}=e^{ix_{2}y_{1}}U_{x_{1}+x_{2},y_{1}+y_{2}},\;x_{j},y_{j}\in R,\;j=1,2.$$

Here, we obtain

$$\left[{\left[\chi \left(g\right)\right]}^{1/2}U_{\chi ,g}\psi \right]\left(g\right)\equiv \left[e^{\frac{ixy}{2}}e^{it\hat{x}}e^{it\hat{p}}\psi \right]\left(t\right)=\left[e^{it\hat{x}+it\hat{p}}\psi \right]\left(t\right),\;x\in \hat{G}≅R,y\in G\equiv R,$$

where $\hat{x}$ and $\hat{p}$ are the standard position and momentum operators [2]. Hence, (9) for this situation is the quantum characteristic function [2]

$$f_{\rho }\left(x,y\right)=Tr\left(\rho e^{ix\hat{x}+iy\hat{p}}\right),\;x,y\in R.$$

The last formula, in turn, allows us to restore the probability distributions introduced in Proposition 2 such that

$$d\mu_{\rho ,\chi ,g}\equiv \omega_{\rho }\left(t,\varphi \right)dt,$$

where

$$\omega_{\rho }\left(t,\varphi \right)=\frac{1}{2\pi }\int_{R}e^{-itr}f_{\rho }\left(r\cos\varphi ,r\sin\varphi \right)dt$$

is an optical quantum tomogram [10,12].

Put

$$\psi_{0}\left(t\right)=\frac{1}{\pi^{1/4}}exp\left(-t^{2}/2\right)$$

**Remark 3.**  *Note that $U_{x,y}\psi_{0}$ is a coherent state corresponding to the complex parameter $\alpha =\frac{-y+ix}{\sqrt{2}}$ and after such a change of variables we obtain $D\left(\alpha \right)=e^{\frac{ixy}{2}}U_{x,y}$ (the displacement operator) [19].*

Taking into account

$$\frac{1}{2\pi }\int_{R}e^{it(x-y)}dt=\delta \left(x-y\right)$$

we obtain that the Haar measure corresponding to the Pontryagin duality [10] is $\frac{1}{2\pi }dx$ and we are coming to the famous Glauber–Sudarshan measure

(12) $$M\left(B\right)=\frac{1}{2\pi }\int_{B}|U_{x,y}\psi_{0}\rangle \langle U_{x,y}\psi_{0}|dxdy,$$

where *B* runs measurable subsets of $R^{2}$. Let us calculate (11) appearing in Theorem 1 for (12).

**Proposition 3.** 
$$T_{x,y}=exp\left(-\frac{x^{2}+y^{2}}{4}\right)e^{\frac{ixy}{2}}U_{x,y},\;x,y\in R.$$

**Remark 4.**  *Thus, the function f(χ,g)≡f(x,y) defined in Theorem 1 is found for the case G=R.*

**Proof.**  Following from (11),

$$T_{x,y}=\frac{1}{2\pi }\int_{R^{2}}e^{iry}e^{-ixt}|U_{r,t}\psi_{0}\rangle \langle U_{r,t}\psi_{0}|drdt.$$

Let us take a change of variables, i.e., $\alpha =\frac{-y+ix}{\sqrt{2}}$ and $\beta =\frac{-t+ir}{\sqrt{2}}$. Then,

$$T_{\alpha }=\frac{1}{\pi }\int_{R^{2}}e^{2iIm(\alpha \bar{\beta })}|\beta \rangle \langle \beta |d^{2}\beta .$$

On the other hand, using the representation of the displacement operator in the form where the creation and annihilation operators $a^{†},\;a$ [19] are used

$$D\left(\alpha \right)={exp(|\alpha |}^{2}/2)exp\left(-\bar{\alpha }a\right)exp\left(\alpha a^{†}\right)$$

we obtain

$$\frac{1}{\pi }{exp(|\alpha |}^{2}/2)exp\left(-\bar{\alpha }a\right)\int_{C}|\beta \rangle \langle \beta |d^{2}\beta exp\left(\alpha a^{†}\right)=$$

$${exp(|\alpha |}^{2}/2)\int_{C}exp\left(2iIm\left(\alpha \bar{\beta }\right)\right)|\beta \rangle \langle \beta |d^{2}\beta .$$

Hence,

$$T_{\alpha }={exp(-|\alpha |}^{2}/2)D\left(\alpha \right).$$

It remains to note that $D\left(\alpha \right)=e^{\frac{ixy}{2}}U_{x,y}$ [19]. □

## 6. Discussion

In this paper, we investigated a projective unitary representation of a direct product $\hat{G}\times G$, where *G* is a locally compact Abelian group and $\hat{G}$ is its dual consisting of characters. Earlier, it was shown [10,14] that orbits of this representation determine a POVM in its closed linear envelope using the Pontryagin duality. Continuing this study, we show that the representation $\left(\chi ,g\right)\to U_{\chi ,g}$ is irreducible. Thus, the obtained POVM is complete in the entire space. Based upon this approach, we construct a family of probability distributions named by us as a set of quantum tomograms that give the alternative characteristics of a quantum state with respect to the density operator ρ. Using the Naimark dilation, we consider a projection-valued density *E* corresponding to our POVM M on the group $G=\hat{G}⊗G$. This measure *E* determines a family of unitary operators $W_{\chi ,g}$ which form a unitary representation of G. By reducing the unitary operators $W_{\chi ,g}$, we introduce a family of contractions $T_{\chi ,g}$ associated with the constructed POVM. It is proven that these contractions are multiples of unitary operators of the representation $U_{\chi ,g}$. This fact allows us to prove the information completeness of the measure M. The conversion formula restoring the state from the probability distribution on $\hat{G}\times G$ is obtained. In Section 6, as an example, the case when G=R and the corresponding POVM is the Glauber–Sudarshan measure determined by projections on coherent states is analyzed.
